# Innovations in Dementia Care in Norway

**DOI:** 10.1093/geroni/igaf122.2580

**Published:** 2025-12-31

**Authors:** Brooke Hollister, Bjørnulf Arntsen, Markus Frydenlund

**Affiliations:** Center for Care Research, south at the University of Agder, Arendal, Aust-Agder, Norway; University of Agder, Grimstad, Aust-Agder, Norway; University of Agder, Grimstad, Aust-Agder, Norway

## Abstract

With a growing population of people with dementia (PWD), a national health care system, and a healthy economy, Norway has the privilege of implementing innovative interventions for PWD. While this reality allows for innovation, policymakers and researchers alike struggle with funding and designing evaluations of complex interventions for PWD. Using guidelines from the Medical Research Council’s Guidelines for developing and evaluating complex interventions and resources from the National Institute on Aging’s IMPACT Collaboratory, researchers in Norway have taken a pragmatic approach to evaluating innovations in dementia care. Two project evaluations were designed with the aim of, 1) adequately describing complex interventions in practice; 2) conducting a process evaluation of implementation processes; 3) assessing potential outcomes on PWD, their caregivers, and health care personnel; and 4) mobilizing knowledge into policy and practice change. Evaluation of the Grimstad Dementia School, which takes a life-long learning approach to a day program for PWD in early to moderate stages, showed promising results, leading to the development and dissemination of Implementation Guidelines for replication in other Norwegian municipalities. The second innovation focused on several Dementia Villages in Norway participating in national and European networks of dementia villages and collaborating researchers. In Norway, this collaboration has resulted in the development of a Program Theory of Dementia Villages as well as several pragmatic evaluations of implementation processes and outcomes. Methods and findings from these studies will presented, in addition to lessons learned regarding replicability and dissemination in both the Norwegian and international context.

